# Supplementary material for: Structural basis for the inhibition of RecBCD by Gam and its synergistic antibacterial effect with quinolones
Source: eLife. 2016 Dec 23;5:e22963. doi: 10.7554/eLife.22963 (PMC5218532; doi:10.7554/eLife.22963)
Supplement: Figure 1—source data 1. — DOI: http://dx.doi.org/10.7554/eLife.22963.003 [file elife-22963-fig1-data1.docx]

**Figure 1 – Source data 1** – EM data statistics and Final model

|  | **GamBCD** |
| --- | --- |
| **Data collection** |  |
| Voltage (KV) | 300 |
| Detector | Gatan K2 |
| Pixel Size (Å/Pixel) | 1.34 |
| Total Dose (e^-^/Å^2^) | 36 |
| Movie Frames | 25 |
| Dose Rate (e^-^/Pixel/s) | 6.5 |
| Defocus Range (μm) | -0.6 to -2.9 |
| **Refinement** |  |
| Final Particles | 122796 |
| Resolution (Å) | 3.8 |
| CC (whole map) | 0.859 |
| CC (around atoms) | 0.817 |
| Clash Score  R.m.s. deviations | 6.6 |
| Bond lengths (Å) | 0.00 |
| Bond angles (°) | 0.48 |
| Ramachandran  Favoured (%)  Outliers | 95.2  3/2996 |
| Rotamers  Favoured (%)  Outliers | 95.0  29/2562 |
